# Global Motion-Aware Robust Visual Object Tracking for Electro Optical Targeting Systems

**DOI:** 10.3390/s20020566

**Published:** 2020-01-20

**Authors:** Byeong Hak Kim, Alan Lukezic, Jong Hyuk Lee, Ho Min Jung, Min Young Kim

**Affiliations:** 1 School of Electronics Engineering, Kyungpook National University, Daehakro 80, Daegu 41566, Korea or byeonghak81.kim@hanwha.com (B.H.K.); leewer354@knu.ac.kr (J.H.L.); ytr789@knu.ac.kr (H.M.J.); 2 Hanwha Systems Co., 1gongdanro, Gumi 39376, Korea; 3 Faculty of Computer and Information Science, University of Ljubljana, 1501 Ljubljana, Slovenia; alan.lukezic@fri.uni-lj.si; 4 Research Center for Neurosurgical Robotic System, Kyungpook National University, Daehakro 80, Daegu 41566, Korea

**Keywords:** visual object tracking, camera motion, motion aware, robust tracking

## Abstract

Although recently developed trackers have shown excellent performance even when tracking fast moving and shape changing objects with variable scale and orientation, the trackers for the electro-optical targeting systems (EOTS) still suffer from abrupt scene changes due to frequent and fast camera motions by pan-tilt motor control or dynamic distortions in field environments. Conventional context aware (CA) and deep learning based trackers have been studied to tackle these problems, but they have the drawbacks of not fully overcoming the problems and dealing with their computational burden. In this paper, a global motion aware method is proposed to address the fast camera motion issue. The proposed method consists of two modules: (i) a motion detection module, which is based on the change in image entropy value, and (ii) a background tracking module, used to track a set of features in consecutive images to find correspondences between them and estimate global camera movement. A series of experiments is conducted on thermal infrared images, and the results show that the proposed method can significantly improve the robustness of all trackers with a minimal computational overhead. We show that the proposed method can be easily integrated into any visual tracking framework and can be applied to improve the performance of EOTS applications.

## 1. Introduction

Visual tracking is one of the core problems in computer vision. The main task of short term visual tracking is to localize the target in consecutive frames in a video. Recently, visual tracking received much attention from researchers, resulting in significant improvements of the tracking algorithms. These improvements are reflected in the large number of tracking benchmarks [1,2,3,4,5,6,7]. The subfield of visual tracking focuses on thermal infrared (TIR) tracking, which is less developed than the RGB based short term tracking. In this paper, we focus on hybrid RGB + TIR images, which is common in image sensors for electro-optical targeting systems (EOTS). As a matter of fact, many defense and security applications such as helicopters and armored vehicles are integrated with EOTS sub-systems. They have stabilizing inner gimbals to achieve high object detection and tracking performance at night and in dynamic motion environments.

**Figure 1:**
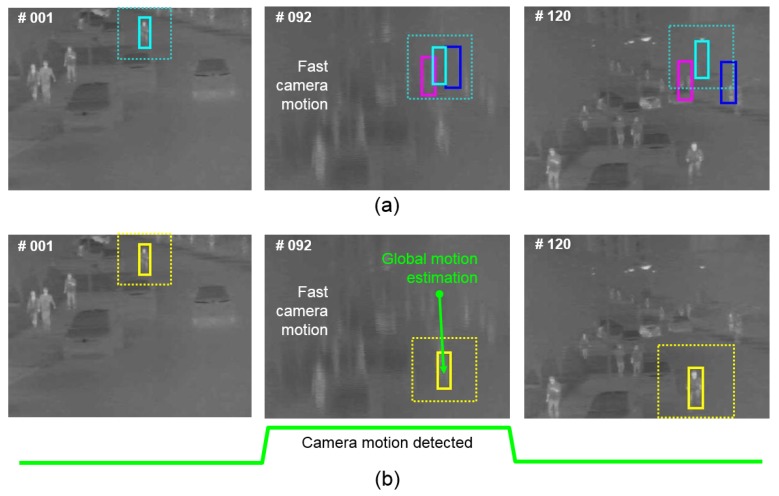
Conventional trackers fail due to the fast camera motion, and they are no longer able to recover the target position (**a**). The proposed tracker can handle large target movements caused by the camera motion and can successfully track the target (**b**).

Thermal infrared images have outstanding advantages over the standard RGB based imaging systems, i.e., the lack of light and reflections is not very problematic in TIR, while on the other hand, there is less color information, which can be useful for robust tracking. These properties result in TIR images having great application potential, especially in surveillance and object tracking missions. A typical tracking scenario in TIR is the tracking of an object that is far; therefore, it is small in the image. Another property is that TIR cameras are static most of the time, but when they are used to search and track to observe a target continuously at a long range at high magnification, the scene moves fast and suddenly, which causes significant camera movement and blurred images. The TIR sensor has a relatively small amount of photon energy compared to the RGB sensor; therefore, its integration time to accumulate photon energy and to make clear images is longer than the integration time of the RGB sensor in the readout integrated circuit (ROIC). TIR images are therefore easily blurred in the event of severe camera movements due to the longer integration time compared to general imaging sensors. As a result of that, trackers on TIR images often fail due to the image blur caused by fast camera motion. Most of the existing trackers do not explicitly address fast camera motion, and they use a fixed sized search region. As shown in Figure 1a, if the target goes out of the search region due to significant camera motion, the tracker fails to keep tracking it continuously. This is more problematic for TIR images, especially in real EOTS applications, since motion blur occurs more often than in RGB tracking due to the sensitivity of the TIR detector and its severe operating environment.

The main contribution of the paper is a new method for global camera motion estimation that can be incorporated into most of the existing tracking algorithms. Figure 1b shows the conceptual idea of the proposed method. We extend four conventional fast correlation filter based trackers with the proposed method, and we show the performance boost in terms of robustness for all compared methods, with a negligible complexity overhead. All methods are tested on the recent Visual Object Tracking Challenge in 2019 (VOT2019) using RGB + TIR datasets, which are not only the most challenging infrared tracking datasets, but also the most similar published infrared datasets with real EOTS applications [8]. Additionally, we analyze extensive experimental datasets on the PTB–TIR and RGB–T234 benchmark [9,10]. Especially, the RGB-T234 object tracking benchmark datasets have in total 234 thermal infrared sequences with 89 fast camera motions in contrast to PTB-TIR, having 17 moderate camera motions. We also apply the proposed tracking method to real EOTS in the conditions of fast camera motions for validation. The results show that the proposed method can improve the robustness of an EOTS and show improvement of the intersection over union (IOU) and center error compared to the conventional tracking methods [11,12].

## 2. Related Works

Correlation filter trackers achieved state-of-the-art results in the visual tracking tasks in recent years. In the following, we review some popular tracking methods based on correlation filters and describe how the field developed in the last few years. The discriminative correlation filter (DCF) methodology was introduced by Hester and Casasent [13]. It was successfully applied to the localization task in visual tracking by Bolme et al. [14], who introduced the minimum output sum of squared error (MOSSE) correlation filter and achieved state-of-the-art results at the remarkable tracking speed of few hundred frames per second. The method was improved by introducing kernels [15] and the multi-channel formulation of DCFs [16]. Danelljan et al. [17] proposed the scale estimation method formulated as a set of one-dimensional correlation filters on each pixel. The advancements and performance improvements of these methods were not only methodological; they also incorporated more complex features such as color names [18], HoG [19] and even CNN based features [3]. Lui proposed a correlation filter based ensemble tracker with multi-layer convolutional features for TIR tracking and found that the features from the convolution layer were more effective than a fully connected layer for thermal infrared tracking [20]. Xin li et al. proposed a TIR tracker via a hierarchical spatially aware Siamese CNN to obtain both spatial and semantic features of the TIR object; the tracker was designed as a Siamese CNN that combined the multiple hierarchical convolutional layers [21]. Li et al. proposed a target aware deep tracking framework integrated with the Siamese CNN and target aware features [22]. Liu proposed a tracker that performed a prediction using a given threshold by providing a template update method as a score function between a candidate group and a template [23,24]. This tracker had an effective observation module, which could deal with occasional large appearance variation or severe occlusions. These state-of-the-art trackers have been successfully researched with various advanced methods to overcome the limitations of conventional trackers using TIR and RGB images. However, in order to consider the application of the embedded tracking module in EOTS, these methods required great computing power with parallel processors, and the processing time was not fast enough, being under 3∼10 fps.

According to the development of deep learning methods, in order to improve tracking accuracy and robustness under the conditions of dynamic objects or camera motions, Danelljan et al. [25] proposed a method of applying a deep RGB feature and a deep motion feature. However, the operational condition with abrupt camera motion caused serious blurred images such that the object and the background could not be distinguished, and as a result, the motion feature map could be expanded and saturated up to the global search area; finally, the coarse localization of the target could not be estimated. Risse et al. [26] proposed a compensation method to find the localization of the target in sequence frames using the RANSAC approach. This method had a limitation with respect o correcting the position of the target in motion blur situations under fast camera motion cases where the target and background are not distinguished. Zhu et al. [27] proposed a framework that worked by using the distractor aware approach to reduce the response of the background rather than the object so that the response score of the object became apparent. However, the semantic negative pairs had a limitation in that they could not find the distractor when fast motion blur occurred. In addition, these methods used pre-trained deep learning models; although their accuracy and robustness were outstanding compared to general DCF based trackers, the accuracy and robustness could be drastically reduced according to the quality of pre-trained datasets, as well as variation of new test environments. Furthermore, it was difficult to apply to real-time EOTS applications since the frame rate was too slow for conventional compact embedded systems. The most useful and high performance object tracking methods for real-time EOTS applications are advanced DCF baseline trackers such as discriminative scale space tracking (DSST) [28].

The following authors made advancements in the size of the search region in correlation filters. Danelljan et al. [29] proposed a method that introduced a penalty function that penalized large filter values far away from the target region. Such a filter can be larger while preventing the background from having a large effect. A similar issue was addressed by Galoogahi et al. [30], who formulated constrained learning within the target region only, and by Lukezic et al. [31], who used a binary mask obtained as color segmentation to constrain the filter. Mueller et al. [5] presented a method, the context aware (CA) correlation filter tracking, to train the correlation filter robustly, by taking into account negative samples. On the other hand, Bertinetto et al. [32] proposed a method (STAPLE) to improve the localization of the correlation filter by combining correlation response and color segmentation in the localization step. All described existing correlation filters localized the target within a search region of a limited size, which was defined by the size of the filter. The search region was centered on the target position from the previous frame. This limitation could cause tracking failure, which could not be recovered in the event of significant camera motion. In a review paper, Li et al. [33] showed that most of the trackers are not guaranteed to function properly with respect to fast camera motions, and in particular, representative DCF trackers, including DSST, showed the most degradation of performance in motion blur (MB) conditions. In this work, we address the fast camera movement issue with a separate background tracking method, which reduces the impact of large displacements between the consecutive frames. We use a motion blur detection method when the camera moves fast, and the global motion is found from the time duration of motion blur frames. To detect motion blur and feature tracking, the motion blur detection method should be reliable and accurate [34]. Moshe Ben-Ezra et al. proposed a motion blur detection method using the hybrid camera system and point spread function (PSF) calculation to detect motion blur and improve the accuracy of the object tracking algorithm [35]. Cho et al. proposed a fast deblurring method that produced a deblurring result from a single image of various sizes in 5∼0.2 fps [36]. The method is general and can be incorporated into any tracking method, as well as DCF trackers. This can especially contribute to improving the robustness of fast DCF based trackers without much additional computational complexity.

## 3. Methods

**Figure 2:**
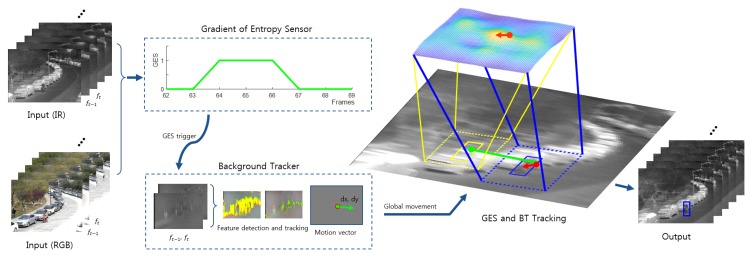
An overview of the proposed method. The IR and RGB images are used in the gradient of the entropy sensor (GES) module to determine when camera motion happens. Then, the background tracking (BT) module is used to estimate global movement on the consecutive IR images. Estimation of the global movement is used to translate the position of the search region.

The EOTS applications with thermal imaging sensors mounted on aircraft, drones, or battle tanks are used under the conditions of static background images because of the long surveillance distance. However, they must perform observation missions under conditions of the complex movement of targets, as well as fast camera motions. Although their background is simpler than general near-field cameras, many of the conventional trackers fail to track the target robustly because the images are significantly blurred. We are motivated to detect this blurring phenomenon using the entropy value and to find the camera fast motion vector effectively to improve the performance of trackers. In this work, we propose a global camera motion estimation framework consisting of the gradient of the entropy sensor (GES) and background tracking (BT), as shown in Figure 2. The GES component (Section 3.1) detects camera motion based on the change of the gradient of the image entropy, and it triggers the BT (Section 3.2) component, which estimates the global fast camera movement.

### 3.1. Gradient of the Entropy Sensor

In the tracking method using TIR images, tracking failure often happens when the images are blurred and the shape of the objects becomes unclear. The image signal processor (ISP) of the thermal image sensor includes a process of setting the integration time for accumulating photon energy due to the low energy level of TIR detectors. Because of the integration time, image blur occurs when fast motion occurs in the camera. In general, the image blur of a thermal image can be detected by the low values of the entropy [37]. We used the phenomenon of motion blur as a sensor signal for fast camera motion detection because the thermal imaging sensors are required to be set with a long integration time and motion blur occurs easily. In general, the motion blur of images is measured quantitatively using the point spread function (PSF) and the discrete entropy (DE) [35]. The PSF derives the level and direction of motion blur as a vector, but requires complex two-dimensional convolution operations. On the other hand, the entropy derives a quantitative value only by the level of motion blur of the image, and the calculation speed is fast due to the simple one-dimensional summation. However, depending on the complexity of the background scenes, the absolute value of the entropy shows large variation. The variation affects the accuracy of the blurring detection and is problematic. Therefore, we propose the gradient of the entropy (GES) method for fast camera motion detection without the variation problems. In summary, the intuition behind GES is a value that can quantitatively detect the amount of change in image blur caused by fast camera motion. Furthermore, GES is described as a sensor signal that detects the timing of fast camera motions for image sequences in real time.

The entropy of information theory was proposed by Claude Elwood Shannon in 1948, which was used as a metric for information chaos [38]. It has been primarily used in data analysis and communication systems to calculate the minimum number of bits required for lossless compression of information. Denoting *A* as a finite set of $M_{A}$ possible states, i.e., $A=\{a_{j}\}$, $j=\{1,\ldots ,M_{A}\}$, the Shannon entropy H(A) is defined as:

(1) $$H\left(A\right)=-\sum_{j=1}^{M_{A}}p\left(a_{j}\right)\;\log\;p\left(a_{j}\right),$$

where $p(a_{j})$ denotes the probability of the state $a_{j}$. In the field of computer vision, the entropy is also used to analyze how well image quality is maintained or improved [39,40]. To obtain the entropy $E_{I}$ of an image *I*, the probability $p(a_{i})$ is changed to $pdf(I_{i})$, calculated as:

(2) $$pdf\left(I_{i}\right)=\frac{|I_{i}|}{n},\;\;\;\;\;i=\left\{0,1,\ldots ,L-1\right\}.$$

The $I_{i}$ represents a specific intensity level in the image, and $|I_{i}|$ is the number of pixels with this intensity. All possible intensity values in the image are denoted as *L*, e.g., in the case of an eight bit grayscale image L=256. *n* is the total number of pixels in the image *I*. The image entropy $E_{I}$ is finally calculated as:

(3) $$E_{I}=-\sum_{i=0}^{L-1}pdf\left(I_{i}\right)\;\log\;pdf\left(I_{i}\right),$$

and it can be used to detect the image blurring caused by camera motion. However, since the sequences of various images have different scenes of different images and have different $E_{I}$ level values, it is difficult to detect the camera motion robustly only by $E_{I}$. Therefore, we calculate the temporal derivative $\Delta E_{I}$ of the image entropy $E_{I}$ as:

(4) $$\Delta E_{I}\left(t\right)=\frac{\partial E_{I}}{\partial t}=\frac{E_{I}^{t}-E_{I}^{t-1}}{t-(t-1)}.$$

The current and previous time steps are denoted as *t* and t−1, respectively, while image entropy at the current and previous time steps is $E_{I}^{t}$ and $E_{I}^{t-1}$, respectively. In general, as an example of using the variation of entropy for motion blur detection, Jiadong et al. proposed a noise robust motion compensation method using parametric minimum entropy optimization [34]. Shuigen et al. proposed a gradient magnitude distribution based no-reference image blur assessment method [41]. Considering visual tracking using two modalities, e.g., thermal infrared (IR) and color (RGB) images, the temporal derivatives of the image entropy are denoted as $\Delta E_{IR}$ and $\Delta E_{RGB}$, respectively. The two modalities are captured using different sensors, which have different optical properties and sensitivities; therefore, they are considered separately to detect camera motion:

(5) $$\begin{array}{cc} GES(t)\;=\; & \left[\Delta E_{IR}\left(t\right)<\alpha_{IR}\right]\wedge [|\Delta E_{RGB}\left(t\right)\left|>\alpha_{RGB}\right]\\ & \wedge [max\left(R\left(t\right)\right)<\tau_{R}] \end{array}$$

The main influential sensitivities of the motion blur are the integration time and the frame rate. The integration time improves the quality of the thermal image and generates blurring [42,43]. GES(t) represents the trigger for camera motion in frame *t*, and $\alpha_{IR}$ and $\alpha_{RGB}$ are sensor specific thresholds, which are adjusted and optimized according to the sensitivity of sensors. In more detail, two parameters are initialized in relation to the integration time and the frame rate. The equations for the initializing parameters are shown in Equation (6). The term of the integration time is also used as the exposed time or the read out time of charged photons. However, the motion blur is also changed depending on the optics, ROIC, image transmissions, and compression processes. Therefore, the final $\alpha_{IR}$ and $\alpha_{RGB}$ values should be fine tuned and verified from the initial values through the ablation test process based on the actual output images.

(6) $$\alpha_{IR}=T_{IR}\times \left(-10^{2}\right),\;\;\;\alpha_{RGB}=T_{RGB}\times \left(0.5\right).$$

where $T_{IR}$ is initialized to the integration time of the IR sensor and $T_{RGB}$ is initialized to the frame rate. For an uncooled type of bolometer as a longer wavelength infrared (LWIR) detector sensor, its integration time is approximated by 600 μs, so the threshold is set to $\alpha_{IR}=-0.06$. For an RGB sensor, its frame rate is 50 Hz (20 ms), so the threshold is set to $\alpha_{RGB}=0.01$. 

**Figure 3:**
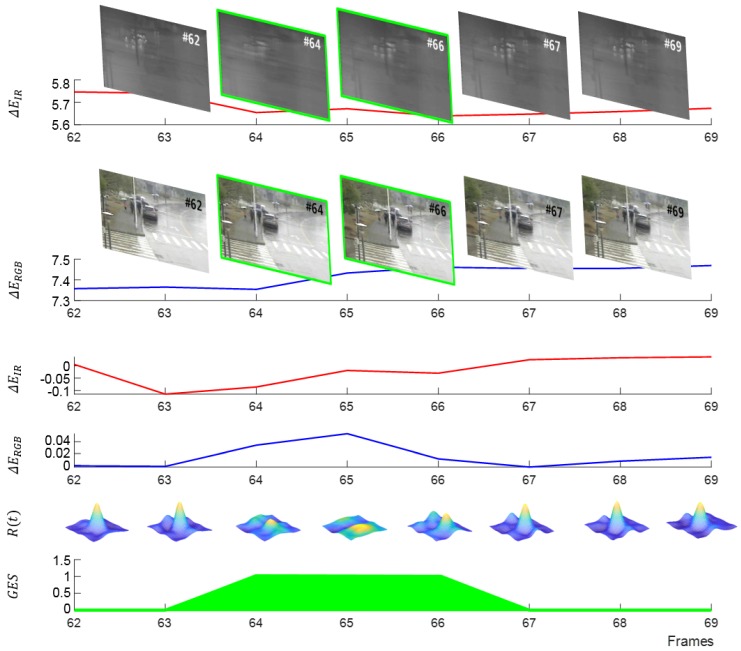
Example of the camera movement detection (camera movement happens between the frames #64 an #66, bordered with green). During that period, $\Delta E_{RGB}$ and $\Delta E_{IR}$ are increased, and *R* is significantly lower, which triggers the camera motion event.

We observed that the correlation response can also be a good indicator to know when the image is significantly blurred. The correlation response of the tracker is denoted as R(t), and the correlation response threshold is $\tau_{R}=0.2$. Figure 3 shows how the GES is calculated on a few consecutive frames where the camera motion occurs.

**Figure 4:**
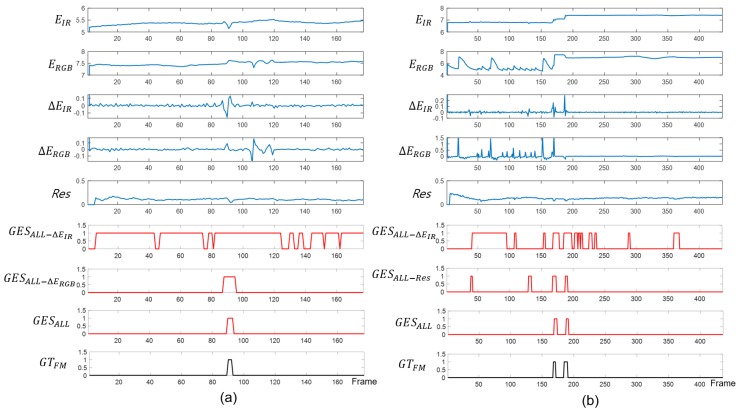
The ablation study of the proposed gradient of entropy method. (**a**) The bikeman sequence and (**b**) cycle3 sequence.

GES is a motion blur detector and is derived from three sub-sensor signals, $\Delta E_{IR}$, $\Delta E_{RGB}$, and Rt, as shown in Equation (5). An ablation study was performed to verify the accuracy of parameter tuning and motion blur detection for the GES optimization, as shown in Figure 4. The ${GES}_{ALL}$ signals are the final detected GES values, which are compared to the ground truth of fast camera motion ${GT}_{FM}$, and they show the results of the corresponding timing when the fast camera motions is occurring. ${GES}_{ALL-\Delta E_{IR}}$ means a result except the $\Delta E_{IR}$ signal; ${GES}_{ALL-\Delta E_{RGB}}$ means a result, except the $\Delta E_{RGB}$ signal; and ${GES}_{ALL-RES}$ shows that it supports removing outlier GES signals. As a result, this ablation study shows that the most accurate performance is derived when the three sub-signals are collaborating altogether.

### 3.2. Background Tracking

**Figure 5:**
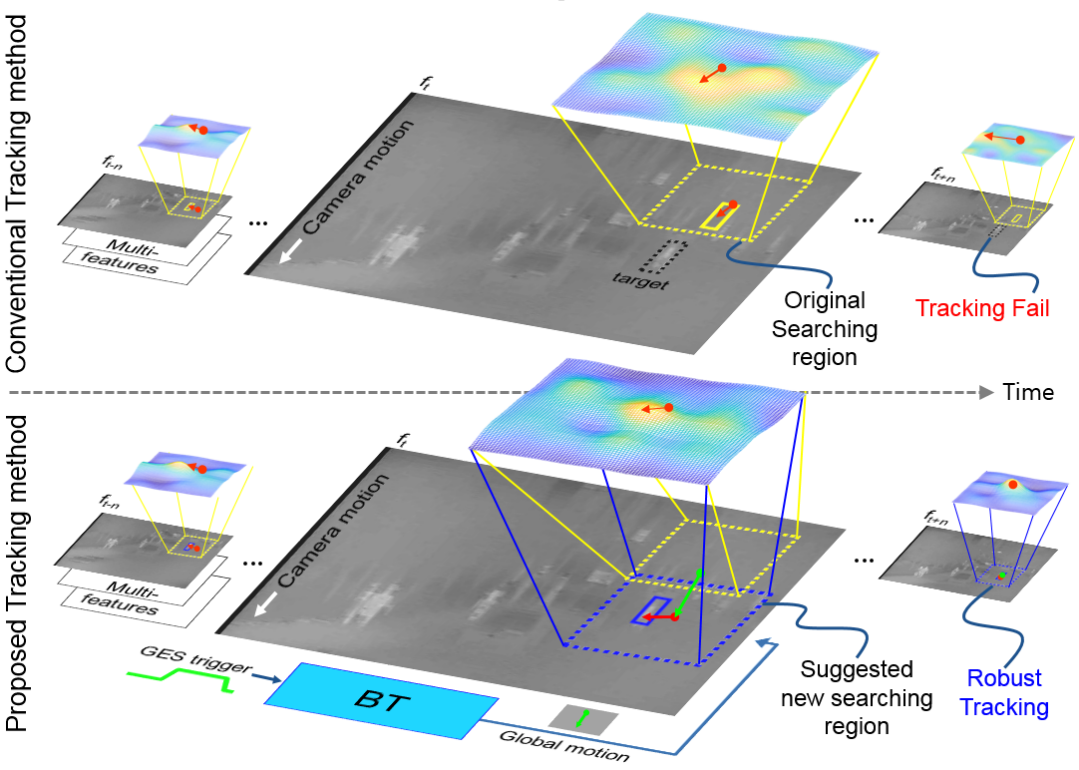
Comparison of the tracking method that does not estimate global camera motion (upper) and the tracker that uses the proposed global camera motion estimation method. The conventional method fails in the event of significant camera motion since the target disappears from the search range. The proposed method estimates the global camera motion and corrects the position of the tracker before localization, which results in successful tracking.

During camera motion, the target position in the image can change significantly, which can cause the target to disappear from the fixed size search range, which makes it impossible for the tracker to localize it. To address this issue, we propose a method to estimate global camera movement and to use it to move the position of the search range of the tracker. The background tracking (BT) framework estimates the translation of the global camera motion from two consecutive frames $f_{t-1}$ and $f_{t}$ when the GES trigger (Section 3.1) detects camera motion. Figure 5 shows the comparison of the proposed tracking method and the conventional tracking method, which does not estimate global camera motion. The BT framework consists of two modules: feature detection and feature tracking.

Feature detection: In the first step of the BT process, the features on the two consecutive frames are detected. The cross-correlation method can be used as a conventional approach to detect the feature of two consecutive image frames and tracking the background. However, when the cross-correlation method is used to detect the background motion of the blurred screen, the convolution calculation of the image has to be performed several times over the entire range, which is very slow. The proposed tracker is required to be fast to be applied to the DCF based trackers of the EOTS in real time. Hence, we employed and modified a feature detector with fast speed and accurate feature point detection performance on the blurry images. Dan et al. performed experiments of four feature detection methods, Shi–Tomasi, Harris–Stephens–Plessey, SUSAN, and FAST [44]. As a result, the Shi–Tomasi method outperformed other methods to detect feature points and showed the fastest speed. Fenghui et al. showed that the Shi–Tomasi method was a more reliable and faster feature detector than other methods for moving IR camera applications [45]. Therefore, for efficient feature detection of the blurred image, we used the Shi and Tomasi corner detection method [46,47], which is an extension of the Harris corner detection method [48]. First, the sum of squared differences (SSD) is calculated using the sliding window approach. The SSD at the position (x,y) in the frame *f* is defined as:

(7) $$SSD\left(\Delta x,\Delta y\right)=\sum_{x}\sum_{y}{\left[f\left(x-\Delta x,y-\Delta y\right)\right]}^{2},$$

where Δx and Δy are the size of the window within which the SSD is calculated. Equation (7) can be linearly approximated as:

(8) $$f\left(x-\Delta x,y-\Delta y\right)\approx f\left(x,y\right)+{\left[f_{x}\left(x,y\right),f_{y}\left(x,y\right)\right]}^{2},$$

where $f_{x}\left(x,y\right)$ and $f_{y}\left(x,y\right)$ are the *x* and *y* image coordinates. The derivation can be further expanded into the following form:

$$\begin{array}{c} SSD\left(\Delta x,\Delta y\right)\approx \sum_{x}\sum_{y}[f\left(x,y\right)\\ +\left[f_{x}\left(x,y\right),f_{y}\left(x,y\right)\right]{\left[\Delta x,\Delta y\right]-f\left(x,y\right)]}^{2} \end{array}$$

and finally written into the matrix form:

(9) $$\left[\begin{array}{c} \Delta x,\Delta y \end{array}\right]\;H\;\left[\begin{array}{c} \Delta x\\ \Delta y \end{array}\right].$$

The score for corner structures is defined as:

(10) $$S_{H}=\lambda_{1}\lambda_{2}-q{\left(\lambda_{1}+\lambda_{2}\right)}^{2},$$

where $\lambda_{1}$ and $\lambda_{2}$ represent the eigenvalues of the matrix *H*.

In the conventional Shi–Tomasi detection algorithm, *q* is a pre-defined constant as a quality threshold of detecting feature points. We observed that it was difficult to determine a single *q* for IR images since they were often poorly textured. Therefore, we proposed to use an adaptive method to select *q* in each frame separately, which we define as:

(11) $$q_{A}=\frac{E_{IR}^{3}}{SF}.$$

Note that $q_{A}$ is an adaptive constant of the *q* in (10), and it is denoted differently just for clarity. $E_{IR}$ denotes the image entropy value (3) of the IR image. The scale factor value (SF) can be adjusted according to the image quality performance parameters including the minimum resolvable temperature difference (MRTD) and modulation transfer function (MTF) performance.

**Figure 6:**
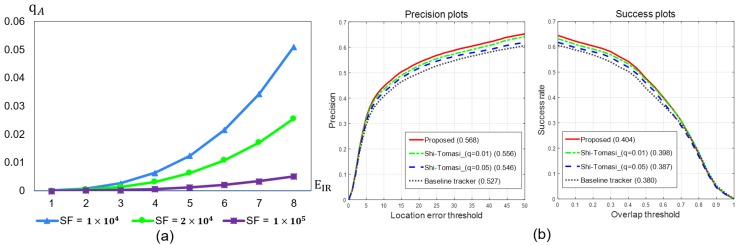
An experimental result for the optimal scale factor value (*SF*) value and the comparison of feature point detectors. (**a**) A comparison of minimum accepted quality values following *SF* and (**b**) different tracking performances by different feature point detecting approaches.

**Figure 7:**
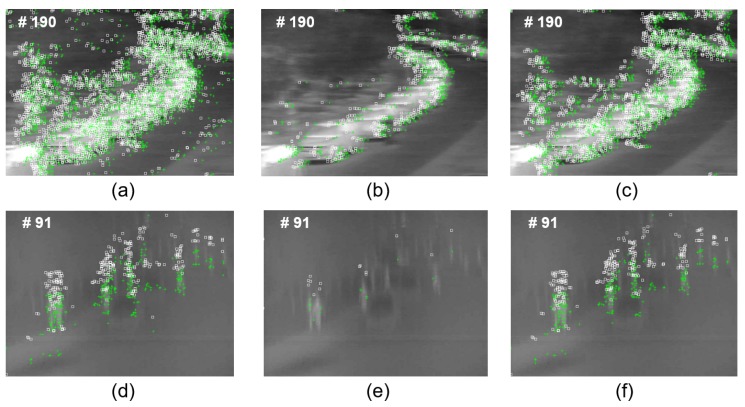
Comparison of different feature point detectors for the background tracking. (**a**,**d**) The Shi–Tomasi detector with a low quality threshold (constant q=0.01). (**b**,**e**) The Shi–Tomasi detector with a high quality threshold (constant q=0.05). (**c**,**f**) The proposed detector (adaptive $q_{A}$ with $SF=2\times 10^{4}$).

We tested several SF values and selected $SF=2\times 10^{4}$. See Figure 6 for the results. The SF value was set such that $q_{A}$ = 0.01 if $E_{IR}=6$, while $q_{A}=0.02$ if $E_{IR}=7$. $q_{A}=0.01$ represents extracting about 1% of the feature points, and $q_{A}=0.02$ reduces the number of feature points by about 0.5%. In Figure 7, there are two scenarios when fast camera motions occurred. The stable number of detected feature points assured the high accuracy of the background tracking. The variation of detected feature points in Figure 7a,d is larger than the proposed detector, Figure 7c,f. As shown in Figure 6b, when different feature point detectors are applied to the baseline tracker, the results showed a quantitative comparison of the precision and the success rate of the trackers. The conventional Shi–Tomasi feature detector had a low quality threshold (constant q=0.01) and a high quality threshold (constant q=0.05), and the proposed tracker had an adaptive $q_{A}$ with the $SF=2\times 10^{4}$ based feature detector. The proposed tracker showed the best performance on both precision and success plots compared to the original feature detecting method. The optimization of the SF value was inversely proportional to the MRTD and MTF performance of the IR camera, which was tuned through experiments.

Kanade–Lucas–Tomasi (KLT) feature matching [49]: Global camera motion between two consecutive frames $f_{t-1}$ and $f_{t}$ is denoted as $d={\left[d^{x},d^{y}\right]}^{T}$. Feature points in frame *t*, denoted as $u_{t}={\left[u_{t}^{x},u_{t}^{y}\right]}^{T}$, can be expressed using feature points from frame t−1 as:

(12) $$\left[u_{t}^{x},u_{t}^{y}\right]={\left[u_{t-1}^{x}+d^{x},u_{t-1}^{y}+d^{y}\right]}^{T}.$$

The KLT method was used to estimate the final camera motion value *d* by repeatedly calculating a point at which a relation between the detected feature points in frame *t* and frame t−1 is minimal.

## 4. Experimental Results

In this section, we present the experimental results of the proposed method for the estimation of global camera motion. We extend four existing DCF based trackers, described in Section 4.1, with the proposed global camera motion estimation module (GESBT) framework. All tested trackers were modified so that GESBT was used before their target localization process. The evaluation methodology of the experimental validation is described in Section 4.2. The quantitative results using VOT2019 RGB-TIR datasets are described in Section 4.3. The qualitative results using VOT2019 RGB-TIR datasets show the performance of the proposed tracker compare with others by visualization in Section 4.4. The quantitative and qualitative results using real EOTS datasets are shown in Section 4.5.

### 4.1. Baseline Trackers

In the experimental comparison, we modified the four existing trackers, described in the following, with the proposed GESBT method. (i) MOSSE_CA [5] is a visual tracking method, which extends the well known MOSSE [14]. It uses simple (grayscale) features only and achieves remarkable robustness. (ii) STAPLE [32] is a combination of the HoG based DSST [28] tracker and a color segmentation method [50]. (iii) STAPLE is further extended with the context aware framework [5] and denoted as STAPLE_CA. (iv) DFPReco [3] is an extension of the ECOtracker [51] by adding part based formulation to the holistic tracker. The main features and release dates of these baseline trackers are shown in Table 1.

### 4.2. Evaluation Methodology

The VOT2019 RGB-TIR dataset (RGB and thermal infrared) was used in all of the experiments consisting of 60 tracking sequences. Since they had several camera motion events, the dataset was used to demonstrate the performance boost of the proposed method. The standard VOT short term reset based methodology [4] was used to evaluate the tested trackers. A tracker was initialized at the beginning of the sequence, and the overlap of the predicted region with the ground truth was calculated in each frame. When the overlap dropped to zero, the tracker was considered as having failed, and it was re-initialized in the following frames. The VOT methodology measures tracking performance using two basic measures: accuracy, which represents average overlap and robustness, which is measured as the average number of failures. The expected average overlap (EAO) is a combination of accuracy and robustness calculated on an average short term tracking sequence. Furthermore, we used the one pass evaluation (OPE) method for extensive experimental validation [6] to demonstrate the effectiveness of the proposed method. All trackers were run on the same workstation (a single Intel CPU i7-7700 3.6GHz 32 GB RAM) using MATLAB.

### 4.3. Quantitative Results

**Figure 8:**
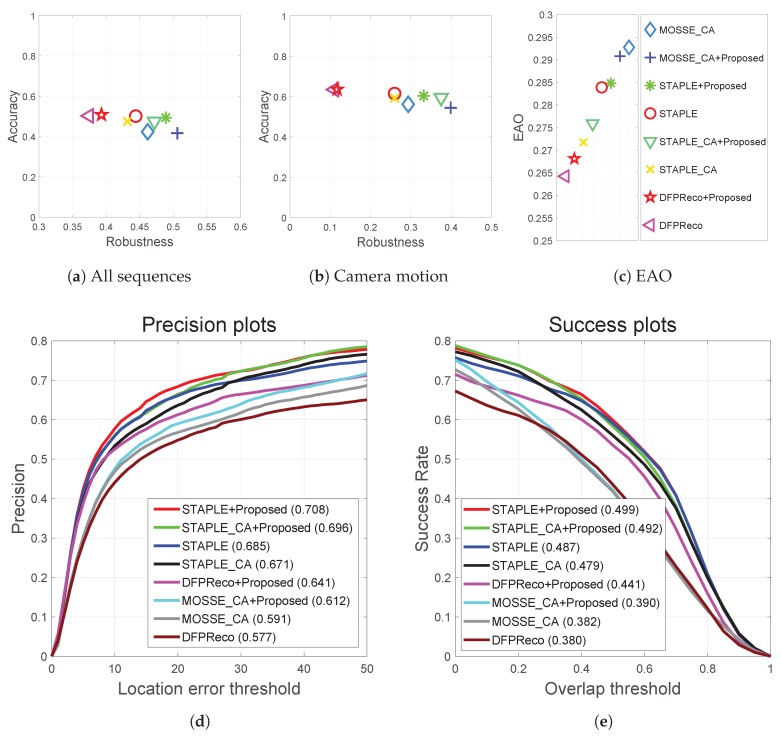
Results of the experimental validation on the Visual Object Tracking Challenge in 2019 (VOT2019)-RGB-TIR datasets. (**a**) An evaluation of the accuracy and robustness for the VOT evaluation, (**b**) the accuracy and robustness under camera motion for the VOT evaluation, (**c**) the expected average overlap (EAO) of the compared trackers for the VOT evaluation, (**d**) the precision plots of the one pass evaluation (OPE) result, and (**e**) the success plots of the OPE result.

The accuracy and robustness of the baseline and modified trackers are shown on the ARgraph in Figure 8a and EAO plot in Figure 8c. The proposed method (GESBT) improved the robustness of all trackers. Table 2 shows the improvements of all methods in terms of robustness. STAPLE_GESBT improved the failure rate of the baseline version by 3.92% by reducing approximately four failures on the whole dataset. The failure rates of MOSSE_CA and DFPReco were improved by GESBT by 2.92% and 2.87%, respectively.

The VOT2019 RGB-TIR dataset had per-frame annotations of the visual attributes, and one of them was camera motion. We compared the trackers under this attribute only and show the results on the AR plot in Figure 8b. The proposed GESBT improved the tracking robustness even further under this attribute, which was an expected result.

**Figure 9:**
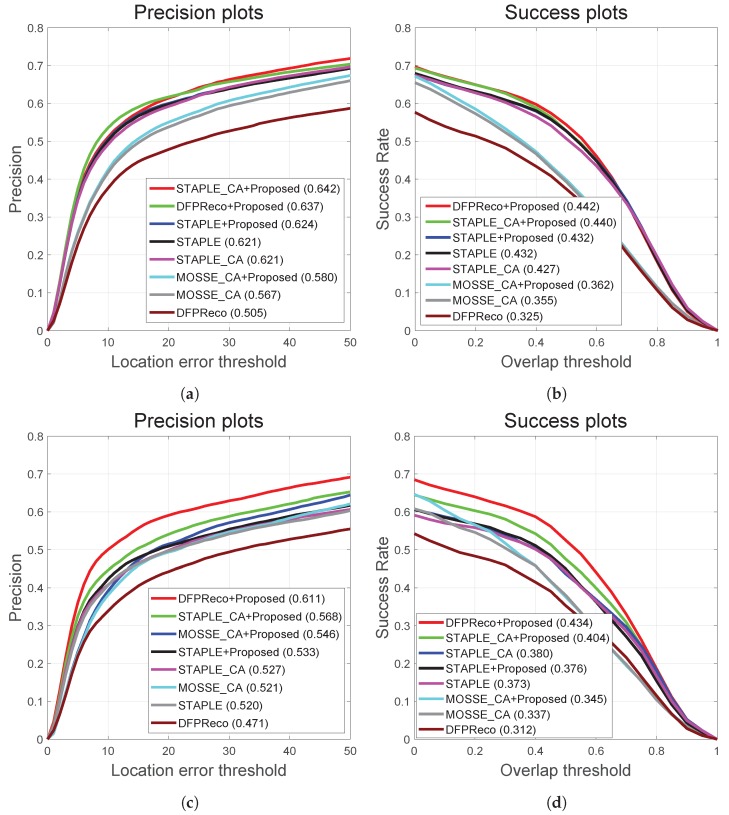
Results of the OPE experiment on the RGB-T234 datasets. (**a**,**b**) are the precision and success plots on the 234 RGB-T234 video sequences, and (**c**,**d**) are the precision and success plots on the 89 RGB-T234 video sequences having fast camera motion.

Additionally, the PTB-TIR and RGB-T234 object tracking benchmarks had various thermal infrared sequences [9,10]. Specifically, RGB-T234 had 89 sequences containing fast camera motion; in contrast, PTB-TIR had 17 sequences with slow camera motions. In order to verify the effectiveness of the proposed method, we carried out extensive experimental validation using the 234 RGB-T sequences and 89 sequences with fast camera motions as shown in Figure 9.

**Figure 10:**
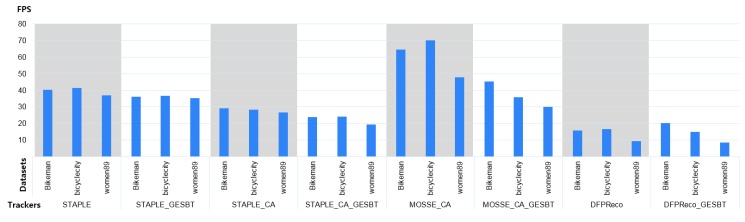
Speed comparison of the baseline trackers and their extensions with the proposed GESBT on three randomly selected sequences from the VOT2019 RGB-TIR dataset. Speed is calculated as frames-per-second (FPS).

Speed analysis: The recently published CA method claimed that the speed was two to six times faster than the compared target-adaptive counterpart (AT) method, and it was shown that it could be applied as half speed (50%) compared to its baseline [5]. Measurements are presented in Figure 10. The proposed method did not significantly reduce the speed compared to the CA method. The average speed for all methods is presented in Table 2. It shows that when CA was applied to the baseline STAPLE, it reduced the speed by 29%, while when GESBT was used, it reduced the speed by 8% only. For more detailed speed analysis, we calculated the speed in frames-per-second (FPS) for three randomly selected sequences from VOT2019 RGB-TIR for all four trackers and their modifications.

### 4.4. Qualitative Results

**Figure 11:**
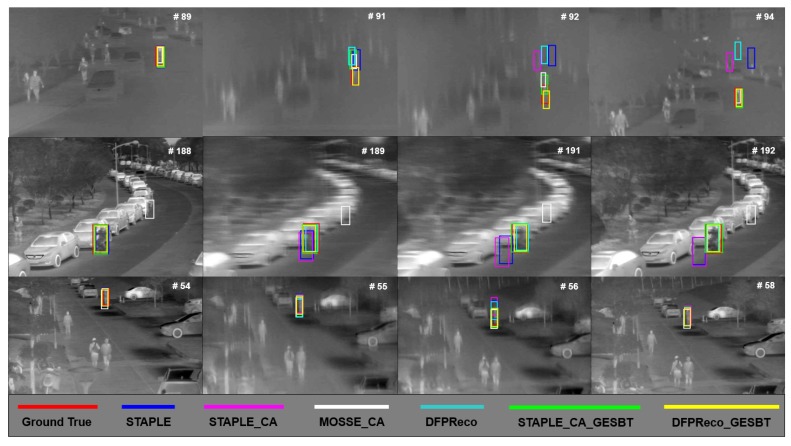
Qualitative examples of baseline trackers and their extension with the proposed method.

Figure 11 shows qualitative results for the baseline trackers and GESBT based extensions of these methods under significant camera motion. In the first two lines, all baseline trackers lost the target in the frame after the camera motion occurred. The trackers with the proposed GESBT were able to track the target successfully even after camera motion. The third line shows that even the baseline trackers were still tracking the target, and the GESBT based methods were tracking the target more accurately.

### 4.5. Results of the EOTS Applications

In this section, we compare the performance of the proposed method with conventional trackers when applied to an EOTS product, which was mounted on an aircraft where actual fast and complex camera motion occurred. There were two main reasons for fast camera motions in aircraft EOTS. First, when the flights or vehicles’ dynamic disturbance exceeded the camera stabilization performance, fast camera motion occurred. In fact, in the case of drastic flying of the aircraft or disturbance of vehicles when they were traveling on uneven roads, the limitation of the tracking performance occurred, and it was recorded in the user operating instructions. Second, the fast camera motion occurred when the camera motor was controlled by the joystick command. The operator tended to keep the target approximately in the center of the image; therefore, significant camera motion could happen when the camera position was adjusted. Such events often resulted in a tracking failure.

**Figure 12:**
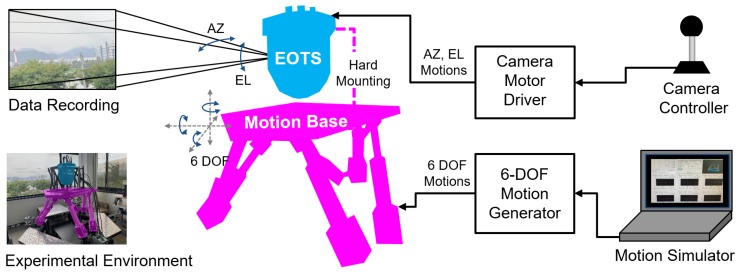
Experimental environment for the data acquisition of an electro-optical targeting systems (EOTS) application.

**Figure 13:**
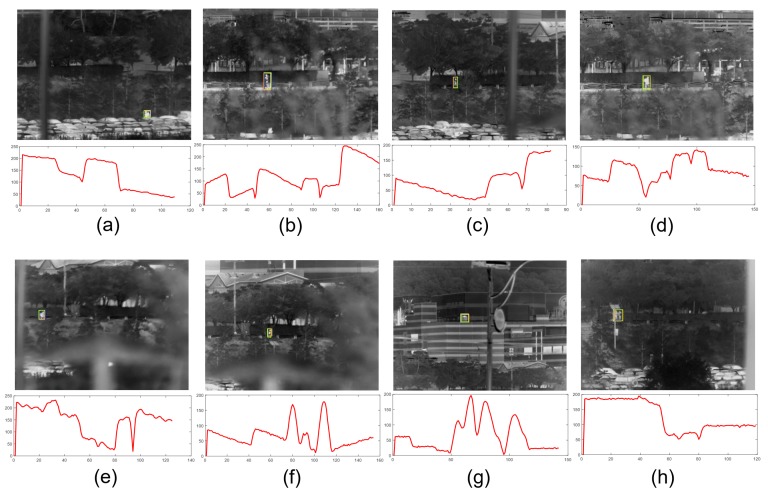
Videos and fast camera motion profiles of a real EOTS application. The solid red lines represent x-axis motion profiles. (**a**–**d**) have fast camera motion caused by the internal camera motor driver; (**e**–**h**) have fast camera motion caused by the external 6-DOF motion generator. The names of the sequences are (**a**) EOTS_parking, (**b**) EOTS_women1, (**c**) EOTS_shortupdown, (**d**) EOTS_clean_man2, (**e**) EOTS_6DOF_bag_cross, (**f**) EOTS_6DOF_up_down, (**g**) EOTS_watertank_free, and (**h**) EOTS_two_roll_updown.

We used the experimental environment as shown in Figure 12 to acquire the video sequences, which included fast motion profiles of the EOTS product. The acquired video sequences included camera motor movement in the azimuth and elevation directions and disturbances in the roll, pitch, and yaw directions applied by the six degrees of freedom (6-DOF) motion simulator. Figure 13 shows examples from the videos obtained by a hard mounted EOTS product in the experimental environment. Figure 13a–d includes the fast motions generated by the movement of the target and the camera motor control. The red solid line in the graph means the Euclidean distance (L2) from the center of the image to the center of the target. Figure 13e–h includes the target motion and the movement generated by the motion simulator. This movement occurred following the pitch, yaw, and roll directions, and the vertical (heave), horizontal (sway), and straight (surge) linear movements were not observed because they were canceled by the parallax at a long distance of more than 200 m.

**Figure 14:**
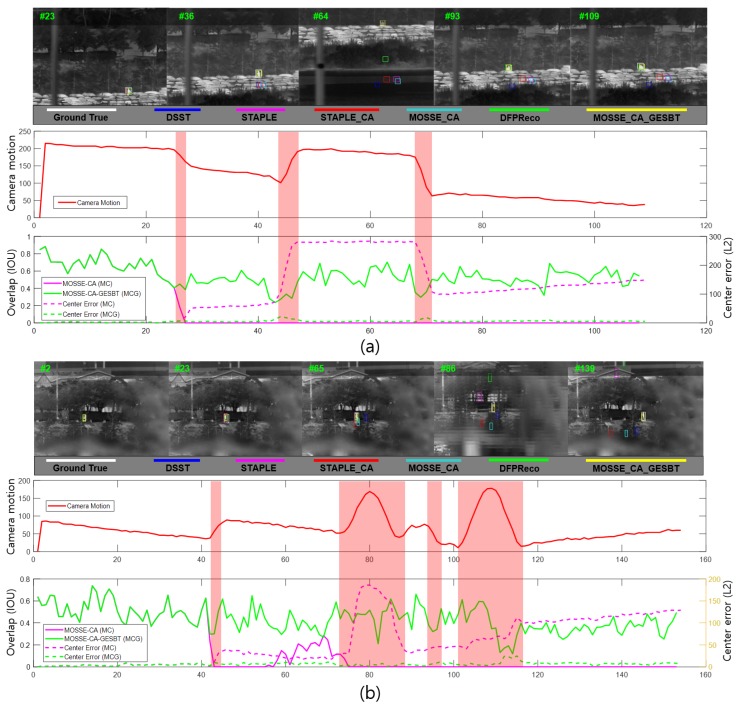
Comparison of the proposed tracker with the conventional tracker. The red solid line on the camera motion graph shows a representative x-axis motion profile. Green and magenta solid lines are the IOU measurement results of the conventional tracker (MOSSE_CA) and the proposed tracker (MOSSE_CA + GESBT). The dotted lines show the center error measurement results of MOSSE_CA and the proposed tracker. (**a**) Tracking result of EOTS_parking dataset; (**b**) tracking result of EOTS_6DOF_up_down dataset.

Figure 14 shows the experimental results for the EOTS_parking and EOTS_6DOF_up_down datasets. The sequential images qualitatively showed continuous object tracking results of the two different trackers. The white bounding boxes represented the ground truth, the yellow boxes the results of the proposed tracker, and others the conventional trackers. In Figure 14a,b, the solid red lines on the graphs show camera motions, and the solid green lines show the intersection over union (IOU) measurement index compared with the conventional tracker by the solid magenta lines [52]. The IOU measurement index function Φ(·) measures the overlap between the region predicted by a tracker and a ground truth region.

As shown in Figure 14, the IOU result of ϕ is calculated between the region of the ground truth $R_{t}^{G}$ and the region of the tracker $R_{t}^{T}$. $\phi_{t}$ is measured until N frames, and it is expressed by Φ($\Lambda^{G}$, $\Lambda^{T}$). In Figure 14a,b, the dotted lines mean the center error calculated by the L2 distance between the center positions of the ground truth and the center of predicted target region by the trackers. The dotted green line is the proposed tracker, and the dotted magenta line is a conventional tracker (MOSSE_CA). All experimental results using other trackers showed improved robustness to track objects continuously. The proposed method especially showed the highest performance improvement ratio when it was applied with MOSSE_CA compared to the others.

## 5. Conclusions

In this study, we proposed a global motion aware method that can be applied to improve the performance of all visual object tracking algorithms for real-time applications. The method consisted of the camera motion detection module based on the gradient of the entropy sensor and the background detection module based on the feature tracking method. Global camera motion was estimated and used in the target localization step. Compared with the existing CA method, the robustness of the proposed method was increased especially when camera motion occurred. The additional computational complexity was very low. We expect that this method will motivate researchers to study the limitations of the trackers in thermal IR based electro-optical systems that are operated in real field environments. Future work includes incorporation of deep CNN features used for estimation of the global motion and formulating the problem as an end-to-end training task.

## Figures and Tables

**Table 1:** Baseline trackers that are extended with the proposed global motion estimation method. The type of features, scale estimation, and year of publication are given for each method.

Trackers	Features	Scale	Published
MOSSE_CA	Grayscale	No	2017 (CVPR)
STAPLE	HoG, RGB histogram	Yes	2016 (CVPR)
STAPLE_CA	HoG, RGB histogram	Yes	2017 (CVPR)
DFPReco	HoG	Yes	2018 (ECCV)

**Table 2:** The improvements of the proposed method compared to the baseline trackers in terms of the number of failures. The red color is the best result, blue the second best, and green the third best.

Method	EAO	Failures	Failure (↓)	Precision (↑)	Success (↑)	FPS
STAPLE	0.28	48.47	Baseline	Baseline	Baseline	39.4
STAPLE_CA	0.27	47.73	0.75	−0.014	−0.008	28.0
STAPLE + Proposed	0.28	44.55	3.92	0.023	0.012	36.1
STAPLE_CA + Proposed	0.27	44.44	4.03	0.011	0.005	22.2
MOSSE_CA	0.29	45.75	Baseline	Baseline	Baseline	55.9
MOSSE_CA + Proposed	0.29	41.83	2.92	0.021	0.008	36.0
DFPReco	0.26	54.43	Baseline	Baseline	Baseline	13.0
DFPReco + Proposed	0.26	51.56	2.87	0.064	0.061	12.8
